# Background Odors Modulate N170 ERP Component and Perception of Emotional Facial Stimuli

**DOI:** 10.3389/fpsyg.2018.01000

**Published:** 2018-06-26

**Authors:** Elmeri Syrjänen, Stefan Wiens, Håkan Fischer, Marta Zakrzewska, Andreas Wartel, Maria Larsson, Jonas K. Olofsson

**Affiliations:** Department of Psychology, Stockholm University, Stockholm, Sweden

**Keywords:** ERP, facial expressions, emotion, odors, N170, LPP

## Abstract

Successful social interaction relies on the accurate decoding of other peoples’ emotional signals, and their contextual integration. However, little is known about how contextual odors may lead to modulation of cortical processing in response to facial expressions. We investigated how unpleasant and pleasant contextual background odors affected emotion perception and cortical event-related potential (ERP) responses to pictures of faces expressing happy, neutral and disgusted facial expressions. Faces were, regardless of expression, rated more positively in the pleasant odor condition and more negatively in the unpleasant odor condition. Faces were overall rated as more emotionally arousing in the presence of an odor, irrespective of its valence. Contextual odors also interacted with facial expressions, such that happy faces were rated as especially non-arousing in the unpleasant odor condition. The early, face-sensitive N170 ERP component also displayed an interaction effect. Here, disgusted faces were affected by the odor context such that the N170 revealed a relatively larger negativity in the context of a pleasant odor compared with an unpleasant odor. There were no odor effects on the responses to faces in other measured ERP components (P1, VPP, P2, and LPP). These results suggest that odors bias socioemotional perception early stages of the visual processing stream. However, effects may vary across emotional expressions and measurements.

## Introduction

Other people, and social relationships, are critical for our survival, and human faces receive preferential processing in dedicated neural networks (Haxby et al., 2000; Palermo and Rhodes, 2007; Rellecke et al., 2013). The ability to infer information about other people is highly advantageous, and such social communication is often non-verbal. Successful social communication primarily relies on accurate decoding of other peoples’ emotional expressions and intentions (Rellecke et al., 2012). For example, the adaptive role of disgust is to initiate behavioral changes that inhibit the entrance of pathogens through the mouth, nose, and skin (Tybur et al., 2009), and disgust is communicated by a distinct facial expression (Rozin et al., 2009; Tybur et al., 2013). Other peoples’ behaviors and facial expressions, therefore, alert us to possible dangers without direct exposure, and the perception of such expressions constitute a remote warning system.

Perception of facial expressions is traditionally investigated by engaging only the visual sense. However, in everyday life, perception of facial expressions is influenced by a multisensory context (Aglioti and Pazzaglia, 2011; Pazzaglia, 2015). Previous research showed that the combination of faces with contextual sounds (Gerdes et al., 2014), body postures (Aviezer et al., 2008), faces embedded within emotional scenes (Righart and de Gelder, 2006, 2008), and preceding emotional pictures, modulate how facial expressions are perceived (Hietanen and Astikainen, 2013). Findings by Hietanen and Astikainen (2013) suggest emotional congruency effects in face-specific ERP components. Contextual effects elicited by odors are less well researched; however, these are of interest because odors are directly propagated by afferent projections to emotionally relevant brain structures (Zald and Pardo, 1997; Soudry et al., 2011). Previous research presents compelling evidence for a close functional association between olfactory and visual areas (Zatorre et al., 2000; Jadauji et al., 2012). In fact, odors may be especially potent emotional triggers (Adolph and Pause, 2012), and arguably less influenced by top–down processes (Ferdenzi et al., 2013). Previous research shows that any odor may increase the speed of behavioral responses in a facial emotion recognition task (Seubert et al., 2010a). Some evidence also indicates that happy expressions are recognized faster in a pleasant odor context (Leppanen and Hietanen, 2003). Both pleasant and unpleasant odor contexts may also enhance recognition accuracy for disgusted faces (Seubert et al., 2010a,b). Furthermore, odors may reduce the amount of emotional information that is needed to recognize a congruent facial expression (Leleu et al., 2015a). Some evidence point to that odor may affect subjective perceptual experiences of faces. For example, Cook et al. (2017) found that odors enhanced the rated valence in an interactive manner (e.g., disgusted faces as more negative in an unpleasant odor context), and that odor valence affected the valence of neutral faces (Cook et al., 2015). However, a recent study showed no odor effects on rated valence or arousal (Syrjanen et al., 2017a). Taken together, some studies suggest that face perception is influenced by a valence-independent odor effect (presumably related to the arousing properties of odors) whereas other studies suggest that emotionally congruent information facilitate facial perception.

Little is yet known about cortical processing that may be involved in these multimodal behavioral interactions, but event-related potentials (ERPs) are well suited to investigate processing stages (Olofsson et al., 2008). Previously it has been shown that faces conditioned with aversive odors differ in how they are processed as early as 50–80 and 130–190 ms after stimulus onset (Steinberg et al., 2012). The P1 component (Rossion, 2014), has been found to be affected by contextual odors in its responsivity to emotional facial expressions in Adolph et al. (2013), however, this effect is not always obtained (Leleu et al., 2015b). Of particular interest in the present study was the N170 component (Rossion, 2014). This component is particularly sensitive to facial expressions (Batty and Taylor, 2003; Trautmann-Lengsfeld et al., 2013; Hinojosa et al., 2015). The inverse of the N170 component, the vertex positive potential (VPP; Rossion and Jacques, 2012), has also been implicated in contextual odor effects (Leleu et al., 2015b). This study found that a pleasant odor increased the amplitude of VPP at the N170 time range regardless of facial expression, consequent exploratory analyses suggested an enhanced amplitude in unpleasant odor conditions at right temporal locations. Thus, odors may influence the processing of faces already at the early stages in the visual stream.

Early ERP components are followed by the mid-latency components P2, N2 and EPN. An enhanced EPN was recently reported for disgusted facial expressions (Hartigan and Richards, 2016). Regarding odor effects at this time range Leleu et al. (2015b) found reduced P2 amplitudes to disgusted and happy relative to neutral expressions in the unpleasant odor condition; this effect was interpreted as the early part of the EPN component. In the N2 time range Cook et al. (2017) found that happy faces in the neutral odor condition were most negative compared to disgusted faces, whereas, in the unpleasant condition the amplitudes for disgusted faces were more negative than for happy faces. However, Rubin et al. (2012) did not find any effects of stress-induced sweat odor at this time interval.

In late stage ERP components, for example, Cook et al. (2017) found congruency effects in the N400 such that happy faces in the unpleasant condition had greater amplitudes than disgusted faces and vice versa. The late positive potential (LPP; Olofsson et al., 2008), has been reported to be sensitive to facial expressions of disgust (Trautmann-Lengsfeld et al., 2013; Leleu et al., 2015b). In one study, human sweat odor collected during anxiety induction was shown to increase participants’ LPP response to neutral and ambiguous facial expressions (Rubin et al., 2012). However, in another study, the LPP response to faces was decreased (Adolph et al., 2013). Lastly, in one study using threat-associated odors, no significant LPP effects were found even though faces were rated as more unpleasant (Kastner et al., 2015). In sum, there is evidence for facial emotion modulation of ERPs at both early and late temporal stages of processing; however, evidence of contextual odor effects is mixed.

In the present study design, neutral baseline conditions allowed the investigation of ERP responses to emotionally congruent combinations of face-odor stimuli, as disgusted faces are most congruent with unpleasant odor, happy faces are most congruent with pleasant odor, and neutral faces are most congruent with no odor. We hypothesized that odors would boost the rated emotionality (valence) and arousal of congruent facial expressions (e.g., happy faces are rated as more happiness-inducing and arousing in a pleasant odor context). Further, we hypothesized that evidence of emotional integration would be observed in the ERP amplitudes. For the P1, we hypothesized only arousing effects of the odors (i.e., higher ERP amplitude in the odor vs. no-odor condition). For the face-selective components such as the N170 and VPP, we hypothesized that odors would lead to increased amplitudes regardless of face valence. Although no previous studies found odor congruency effects on the N170, we hypothesized effects in the N170 time range because of findings using odor conditioning and in studies using visual primes, and considering the close connection between olfactory and visual areas. We hypothesized that odor and emotional expression would interact at the P2 component. For the LPP we hypothesized only main effects of odor.

## Materials and Methods

### Subjects

A total of 60 participants were tested in the lab, but two of these were excluded from the final sample (one participant for task non-compliance and one participant choose to drop out during testing). The final sample thus included 58 participants from a Swedish student population (**Table 1**). None of the participants reported any psychiatric or neurological disorders in a pre-screening protocol, none reported severe and uncorrected deficits in visual or olfactory perception, and none reported severe untreated allergy. After signing the informed consent form, all participants completed the I-PANAS-SF scale (The International Positive and Negative Affect Schedule Short Form; Thompson, 2007), and Karolinska Sleep Questionnaire (KSQ; Nordin et al., 2013). All subjects gave written informed consent in accordance with the Declaration of Helsinki. The protocol was approved by the regional ethics board 2014/2129-31/2.^1^

**Table 1:** Demographics, personality measures and odor concentrations (*N* = 58).

	Mean	*SD*
Age	25.57	5.76
Gender	57% female (*n* = 33)	
Handedness	88.46% right handed	
Years in school	15.36	2.08
Positive affect	29.87	5.92
Negative affect	13.89	4.47
TDDS	26.21	9.02
CSS	32.29	7.37
Valeric acid concentration	33.66	26.43
Lilac concentration	29.72	22.46
Valeric valence	-2.59	2.57
Lilac valence	3.95	2.43

The participants also completed a form regarding demographic information, the Edinburgh handedness scale (Oldfield, 1971), and scales that targeted social and personality variables: the TDDS (Three-Domain Disgust Scale; Tybur et al., 2009), Chemical Sensitivity Scale (CSS; Nordin et al., 2004), see **Table 1**.

### Stimuli

We used face stimuli in color from the FACES database (Ebner et al., 2010). In our selection of pictorial stimuli, 24 actors (6 young women, 6 young men, 6 elderly women and 6 elderly men) expressed three facial expressions (disgust, neutral and happy), this stimulus set consisted in total of 72 unique pictures. The choice of young and old actors was motivated by a desire to generalize our results across adult age. The pictures were selected such that the emotions expressed by each actor were recognized by more than 80% of the participants in a normative sample (Ebner et al., 2010). All of these selected pictures were realigned in the image manipulation software GIMP such that each actor’s eyes were aligned to a common standard. The unpleasant odor stimulus was valeric acid (Sigma–Aldrich), which smells like sweat. A lilac essence (Stockholms Eter & Essencefabrik), which is commonly used in detergent and soap, was used as the pleasant odor stimulus. These two odors were diluted with a nearly odorless solution, Propylene glycol (1,2-propanediol 99%, Sigma–Aldrich) in concentrations of 1%, and 10–100% in steps of 10%.

### Apparatus

Pictures were shown on a 24″ Benq XL2430-B TN-screen with a refresh rate of 100 Hz and a resolution of 1920 × 1080 pixels. Picture size was 9 cm (8.6°) wide and 11.3 cm (10.8°) high. The background was dark gray, viewing distance was 60 cm (maintained with a chin rest), and the experimental software was Presentation 17.0 (Neurobehavioral Systems, Albany, CA, United States).

The EEG apparatus was an Active Two Biosemi system (Biosemi, Amsterdam, Netherlands) with 128 active electrodes. Data were sampled at 512 Hz and filtered with a hardware low-pass filter at 104 Hz.

### Procedure

The main experimental session started by the participants rating the odor stimuli so that both odors should have the same subjective intensity, this procedure was designed to account for individual differences in odor perception. Thus, the participants rated each odorant in 3 concentrations, 1, 40, and 80%, for valence (positive and negative) and intensity on the Borg CR-100 scale (Borg and Borg, 2002), a scale that has been used previously for measuring subtle changes in perceived odor intensity (Olofsson and Nordin, 2004). From the individually obtained intensity ratings, we estimated the odor concentration that matched a moderate intensity (25 on the scale) by using a linear interpolation. For individual subjects, odors could range from 1 to 100% concentration in steps of 10%. In the no-odor control condition we used cotton rolls with only Propylene glycol. Across subjects, mean concentration of the valeric acid used in the experiment was 33.66 (*SD* = 26.43) and the mean concentration of the lilac essence 29.72 (*SD* = 22.46).

The ERP experiment followed the odor rating procedure; the participants were comfortably seated in an armchair in front of a computer screen. They wore an electrode cap for ERP recordings, and their head movements were stabilized by a chinrest. The experiment was divided into six blocks, two with an ambient unpleasant odor, two with a pleasant odor, and two with no odor present. For each subject, block order was random, with the constraint that the same odor could not appear twice in a row. In each block, participants were presented with a series of images of faces. Each face stimulus expressed either disgust, happiness, or a neutral emotional state. For each participant, these pictures were randomly assigned to each block such that each block contained pictures of different individuals. Specifically, each block contained four unique actors expressing each of the three emotions. Each of these 12 pictures was randomly presented 16 times in each block, resulting in 192 trials per block. The odor manipulation was carried out by placing a cotton roll (Pluradent, size 1, about 2 inches long and 1/3 inch radius) inside a cotton tube bandage (Danatube, size 01) that was fixed below the nose of the participant, the ends of the cotton tube were tied together behind the participants head.

**FIGURE 1:**
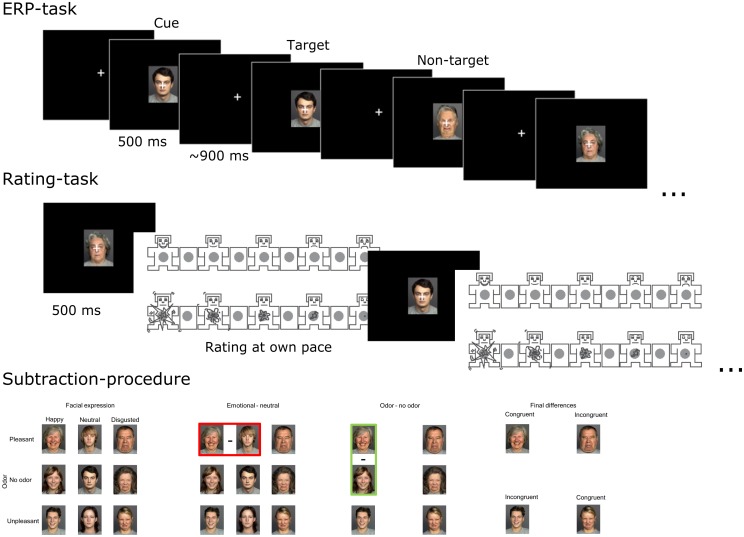
**Top row** depicts the main block with the first two trials illustrating the 1-back task. The **middle row** depicts the picture rating procedure with valence rating at the top and arousal at the bottom. The **bottom row** illustrates our difference-score calculation procedure. Based on the original 3∗3 design **(bottom left panel)**, we first subtracted emotional conditions from neutral conditions **(bottom panel, second from left)** separately for each emotional facial expression (illustrated within the red box for happy - neutral). Then, odor conditions was similarly subtracted from the no odor condition (**bottom panel, third from left**; green box illustrates the subtraction of pleasant from no-odor condition). This resulted in four conditions, two that were congruent (happy face + pleasant odor; disgusted face + unpleasant odor) and two that were incongruent (happy face + unpleasant odor; disgusted face + pleasant odor; bottom panel, fourth from left).

On each trial, a central fixation cross was presented on the screen for between 900 and 1000 ms, then a face was presented for 500 ms (cf. **Figure 1** for a graphical display of the procedure). Participants were instructed to maintain their fixation on the cross. During the experiment, the participants performed a simple one-back task (i.e., a specific picture was repeated) to ensure that participants were attending to the stimuli. One out of twelve stimuli (8.33%) constituted a target trial (i.e., a trial where a picture was repeated). The participants responded by pressing a button to the targets, which were randomly distributed to occur once within every series of 12 stimuli. At the end of each block participants rated how each picture affected them on both valence and arousal dimensions using the standard SAM rating procedure (Lang et al., 2008). Then, odors were removed and replaced, and the participants had the opportunity to rest briefly before the next block. The duration of each block was approximately 5 min and the total testing time approximately 30 min.

### Data-Analysis

We used the R statistical package to extract, preprocess and statistically analyze behavioral data from log files. We used the paired samples *t*-test to statistically test whether the average odor concentrations for valeric acid and lilac differed. We interpolated the rated valence for the individual odor concentrations and used a paired samples *t*-test to statistically test whether the odors differed in the interpolated valence ratings. We calculated the hit rate (only responses within 1 s after stimulus onset was considered) in the 1-back task for each participant to ensure task compliance, and we found that the mean hit rate was 80% (*SD* = 16.34), and the mean false alarm rate was 2% (*SD* = 7.34). We did not further analyze these results as the task was only a means to ensure that the participants attended to the picture stimuli. To assess the overall pattern of the results, we performed a full 3 by 3 repeated measure ANOVA for each measure. Then we tested specific hypotheses regarding congruency effects using difference scores between emotional and neutral stimuli. The aim was to reduce model complexity and aid in interpretation of the results. We calculated difference scores by subtracting the response in neutral from emotional faces. This was followed by subtracting the responses in no-odor conditions from odor conditions for each facial expression (see bottom panels in **Figure 1** for a graphical illustration of the difference score calculation procedure). These scores were subjected to a 2 by 2 repeated measures ANOVA (i.e., conditions with congruent and incongruent odor and face pairs) using the ez-package (Lawrence and Lawrence, 2016) in R. Significant interactions were followed by planned repeated measures *t*-tests between congruent and incongruent conditions. To extract effect sizes (Cohen’s *d* and partial eta squared) and format the output from the statistical tests (*t*-tests and ANOVAs) we used the apa-package in R (Gromer, 2017). We followed up these ANOVAs with pre-planned paired samples *t*-tests to investigate the specific effect of the odors on each facial expression. To counteract the multiple comparison problem, *p*-values were corrected using the Holm–Bonferroni method within each follow-up analysis (Holm, 1979). In ANOVAs that did not meet the assumption of sphericity, we adjusted the degrees of freedom with the Greenhouse–Geisser correction; these are denoted by subscripted GG where applicable in the results section. For all figures, we calculated 95% CIs around the mean, these were corrected as suggested by Morey (2008) to account for within subject designs.

### EEG Data

We preprocessed the EEG data offline with custom Matlab scripts and the FieldTrip (v. 20161002) toolbox (Oostenveld et al., 2011). For each subject, we visually identified noisy electrodes or electrodes that exhibited signal loss and excluded them before the independent component analysis (ICA) analysis. We used best practice methods in the lab to extract ERPs for the ICA analysis. We extracted ERPs only for face pictures that did not require a response. ERPs were derived from the continuous data 200 ms prior to picture onset until 1 s after, re-referenced to Fz, down-sampled to a 250 Hz sampling rate, high-pass filtered at 1 Hz, and baseline corrected. We then performed the ICA analysis on these ERPs using the runica implementation available in FieldTrip. Eye-blink artifacts were identified in the resulting ICA-components by visual inspection of topographies and artifact time-courses, which were compared with a previously computed virtual EOG-electrode consisting of the 5-foremost frontal EEG-electrode. These identified eye-blink components were then rejected and the trials were reconstructed excluding eye-blink artifacts. Then, noise-prone electrodes (*M* = 9.22, *SD* = 5.32 per subject) were interpolated by calculating neighboring electrodes with the triangulation method, and interpolated with the spherical splines method. Epochs were average referenced, baseline-corrected, and low-pass filtered at 30 Hz with the default settings in FieldTrip. Lastly, the trials where the mean amplitude in a channel or trial in comparison with other channels and trials exceeded 3 *z*-scores or had an amplitude range exceeding 100 μV were rejected. After rejecting trials with artifacts, the mean number of trials in each of the 9 conditions was 105.61 (*SD* = 14.69).

Relevant ERP components were visually identified (with guidance from previous literature for each component) from grand mean waves and topographies. To avoid concerns about multiple implicit comparisons (see discussion in: Luck and Gaspelin, 2017), we used the grand averaged ERP-waves across subjects and conditions to identify each specific component. Figures containing topographies, ERP waves, and means for non-significant odor effects in ERP components are included in the Supplementary Material. Although the P1 has been shown to be sensitive to low-level properties of the stimulus (Rossion, 2014), our odor-based contrasts would be unaffected by such visual stimulus differences and we thus included the P1 in our analyses, enabling comparison with previous research (Adolph et al., 2013; Leleu et al., 2015b). The P1 component was apparent in occipital electrodes (16 electrodes around O1 and O2 electrodes according to the 10–10 system), at 120–160 ms after stimulus onset. Although, Leleu et al. (2015b) found effects in the left side N170 component, in the present study the component was more pronounced at right temporal electrodes as a negative deflection, we confirmed with an ANOVA that there was no statistically significant hemispheric interactions (6 electrodes near P8, see **Figure 3**) at 160–200 ms after stimulus onset (patterns consistent as in: Trautmann-Lengsfeld et al., 2013; Zhang et al., 2014). We also, similarly as Leleu et al. (2015b), tested the inverse of the N170 component, Vertex Positivity Potential (VPP), at central sites (10 electrodes around Cz) in the same time frame as the N170, 160–200 ms after stimulus onset (for the VPP component we used a linked mastoid reference using the D32 and B10 electrodes). For comparability with previous research, we also included the P2 in the analysis, which was identified as an occipital positivity (6 electrodes around O1 and O2) 220–300 ms after picture onset. The LPP was identified as a sustained positivity in parietal-occipital electrodes (18 electrodes around Pz) at 300–500 ms after stimulus onset (similar as: Rubin et al., 2012; Adolph et al., 2013). For all components, we computed the mean amplitudes across the relevant electrodes and time range for each condition and participant. To investigate the effects of odor and expression on ERP components, we followed the same analysis strategy as for odor ratings, we performed 3 by 3 repeated measures ANOVAs and then computed difference scores between affective and neutral stimuli on which we performed 2 by 2 repeated measures ANOVAs. To aid in interpreting the results, means and SDs for each measure and condition is reported in **Table 2**, to reduce clutter, the figures for the P1, VPP, P2, and LPP is in the Supplementary Materials. To further the cumulative progress of science and help in establishing reproducible results and aid in future meta-analyses we provide full experimental data at following url: https://figshare.com/s/252745f56a0f7b7115fc (Syrjanen et al., 2017b).

**Table 2:** Mean and (*SD*) for each condition and measure.

Odor/Expression	Valence	Arousal	P1	N170	VPP	P2	LPP
Pleasant Happy	6.78 (0.73)	4.83 (1.47)	4.32 (1.88)	−0.54 (2.21)	1.96 (4.16)	4.90 (2.73)	2.42 (1.53)
Pleasant Neutral	4.64 (0.41)	3.64 (1.30)	4.25 (1.96)	−0.59 (2.17)	2.10 (4.09)	4.89 (2.83)	2.46 (1.66)
Pleasant Disgusted	3.49 (0.80)	4.23 (1.49)	4.27 (1.94)	−0.70 (2.19)	2.12 (4.08)	4.76 (2.74)	2.41 (1.58)
Neutral Happy	6.74 (0.76)	4.62 (1.57)	4.31 (2.01)	−0.55 (2.29)	1.84 (4.24)	4.81 (2.85)	2.47 (1.66)
Neutral Neutral	4.59 (0.45)	3.43 (1.32)	4.36 (1.90)	−0.57 (2.22)	2.00 (4.16)	4.84 (2.89)	2.52 (1.67)
Neutral Disgusted	3.49 (0.85)	4.05 (1.43)	4.26 (2.02)	−0.61 (2.25)	1.88 (4.24)	4.73 (2.94)	2.41 (1.69)
Unpleasant Happy	6.63 (0.84)	4.49 (1.49)	4.34 (1.88)	−0.57 (2.06)	2.20 (4.00)	4.74 (2.62)	2.49 (1.64)
Unpleasant Neutral	4.52 (0.51)	3.62 (1.34)	4.35 (1.92)	−0.60 (1.96)	2.18 (4.04)	4.70 (2.69)	2.44 (1.59)
Unpleasant Disgusted	3.35 (0.73)	4.27 (1.54)	4.25 (1.97)	−0.47 (2.15)	2.10 (4.15)	4.64 (2.68)	2.45 (1.63)

*Valence and arousal on a nine point scale and the ERP-components in μV.*

## Results

Demographic data, the three domains of disgust scale, chemical sensitivity scale, and odor ratings are reported in **Table 1**.

### Odors

A paired samples *t*-test showed that the mean selected concentrations of the valeric acid did not differ from the lilac concentrations (see **Table 1** for mean and SD); *t*(57) = 1.00, *p* = 0.32. As expected, a *t*-test for the interpolated odor valence between valeric acid and lilac showed that these ratings were more negative for the valeric acid odor than for the lilac odor, *t*(57) = −12.52, *p <* 0.001 (see **Table 1** for mean and SD).

### Valence

**FIGURE 2:**
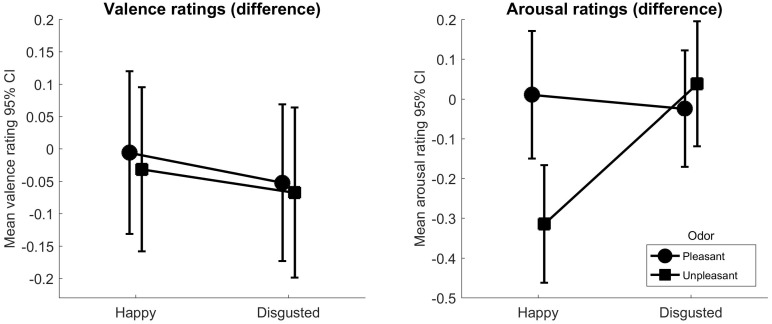
The panels show the rated valence **(left)** and arousal **(right)** difference scores for pleasant and unpleasant odors, separately for happy, and disgusted facial expressions (means and 95% confidence intervals for difference scores. The 95% CIs around the means were corrected as suggested by Morey (2008) to account for within subject designs.

The interaction between odor and expression for the valence ratings in a 3 × 3 repeated measures ANOVA (pleasant, no odor and unpleasant odor vs. happy, neutral, and disgusted expressions) was not significant, *F*(4, 228) = 0.20, *p* = 0.94, ${\text{η}}_{\text{p}}^{2}$ < 0.01. However, there was a main effect of odor, *F*(2,114) = 5.80, *p* = 0.004, ${\text{η}}_{\text{p}}^{2}$ < 0.09, indicating that the faces were rated as more negative overall in the unpleasant odor condition compared to pleasant, *t*(173) = −3.62, *p* < 0.001, *d* = −0.27, and no-odor conditions, *t*(173) = −2.76, *p* = 0.006, *d* = −0.21. Faces in the pleasant odor condition compared to no-odor did not differ in a statistically significant manner, *t*(173) = 0.97, *p* = 0.333, *d* = 0.07. Lastly, as expected there was a main effect of expression, *F*(2,114_GG_) = 348.73, *p* < 0.001, ${\text{η}}_{\text{p}}^{2}$ = 0.86. Happy faces were rated as more positive than neutral, *t*(173) = 33.39, *p* < 0.001, *d* = 2.52, and disgusted faces were rated as more negative than neutral faces, *t*(173) = −17.81, *p* < 0.001, *d* = −1.35. We investigated specific congruency effects on the difference scores (left panel in **Figure 2**), there was no statistically significant interaction or main effects on the 2 × 2 ANOVA, (all *F*s < 0.29, *p*s > 0.591).

### Arousal

A 3 × 3 ANOVA on arousal ratings showed that the interaction between odor and expression was statistically significant, *F*(4,228) = 5.40, *p* < 0.001, ${\text{η}}_{\text{p}}^{2}$ = 0.09. Further, the main effect of odor was significant, *F*(2,114) = 40.56, *p* = 0.024, ${\text{η}}_{\text{p}}^{2}$ = 0.06. Faces in the pleasant condition were rated as more arousing than in the no-odor condition, *t*(173) = 4.15, *p* < 0.001, *d* = 0.31. However, there was no statistically significant difference between the pleasant and unpleasant condition, *t*(173) = 1.81, *p* = 0.072, *d* = 0.14, and unpleasant and no-odor conditions, *t*(173) = 1.67, *p* = 0.096, *d* = 0.13. Lastly the main effect of expression was also significant, *F*(2,114) = 2.72, *p* < 0.001, ${\text{η}}_{\text{p}}^{2}$_=_ 0.42. We tested odor and facial congruency effects on the difference scores with a 2 × 2 ANOVA (right panel in **Figure 2**), the results were significant for the interaction, *F*(1,57) = 12.39, *p* < 0.001, ${\text{η}}_{\text{p}}^{2}$ = 0.18. Specific *t*-tests showed that happy faces in the unpleasant odor condition were rated as less arousing than in the pleasant odor condition, *t*(57) = −2.92, *p* = 0.005, *d* = −0.38. The arousal ratings of disgusted faces were not affected by the odor, *t*(57) = 0.57, *p* = 0.569, *d* = 0.07.

### P1

We approached the P1 component of the ERP response with a similar analytical strategy as for the behavioral data. We performed a 3 × 3 (odors by expressions) repeated measures ANOVA, and none of the effects were significant. For the interaction, *F*(4,228_GG_) = 1.30, *p* = 0.277, ${\text{η}}_{\text{p}}^{2}$ = 0.02; for the main effect of odor, *F*(2,114) = 0.11, *p* = 0.898, ${\text{η}}_{\text{p}}^{2}$ < 0.01, and for the main effect of emotional expression, *F*(2,114) = 0.97, *p* = 0.384, ${\text{η}}_{\text{p}}^{2}$ = 0.02. We investigated specific congruency effects with a 2 × 2 ANOVA on the difference scores, the test did not reach significance, for the odor by expression interaction, *F*(1,57) = 3.28, *p* = 0.075, ${\text{η}}_{\text{p}}^{2}$ = 0.05; main effect of odor, *F*(1,57) = 1.78, *p* = 0.187, ${\text{η}}_{\text{p}}^{2}$ = 0.03, and main effect of expression, *F*(1,57) = 0.02, *p* = 0.887, ${\text{η}}_{\text{p}}^{2}$ < 0.01.

### N170

**FIGURE 3:**
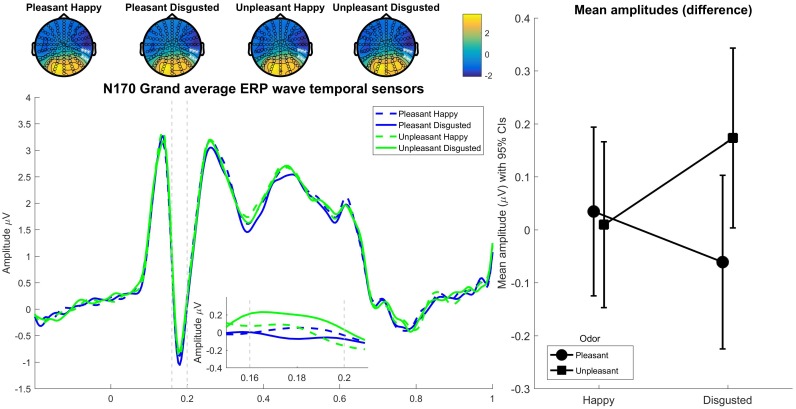
The **(top row)** shows the raw topography of grand average (160–200 ms from stimulus onset) N170 for each odor-face condition. The **(left)** shows grand average ERP waves (of right temporal sensors, highlighted in the **top row** topographies) in each condition. The insert zooms in at the difference waves between emotional and neutral conditions in the N170 peak. The **(right)** shows mean amplitudes and 95% confidence intervals (corrected as per Morey (2008) to account for within subject designs) around the mean amplitude difference between emotional and neutral conditions.

We analyzed the right-dominant N170 component with similar methods as previous measures. The 3 × 3 ANOVA showed no significant interactions between odor and expression *F*(4,228) = 1.76, *p* = 0.139, ${\text{η}}_{\text{p}}^{2}$ = 0.03. The main effects test of odor did not reach significance *F*(2,144_GG_) = 0.45, *p* = 0.619, ${\text{η}}_{\text{p}}^{2}$ < 0.01, nor did the main effect for expression *F*(2,114) = 0.34, *p* = 0.714, ${\text{η}}_{\text{p}}^{2}$ < 0.01. We tested specific odor and facial congruency effects with a 2 × 2 ANOVA (see **Figure 3** that show difference scores, and topographies and ERP waves for each condition of interest), and we found that the interaction was significant, *F*(1,57) = 4.38, *p* = 0.041, ${\text{η}}_{\text{p}}^{2}$ = 0.07. Neither the main effect of odor, *F*(1,57) = 2.33, *p* = 0.133, ${\text{η}}_{\text{p}}^{2}$ = 0.04, nor expression, *F*(1,57) = 0.00, *p* = 0.979, ${\text{η}}_{\text{p}}^{2}$ < 0.01, reached significance. Specific *t*-tests showed that the N170 amplitude for disgusted faces in the pleasant odor condition was more negative than in the unpleasant odor condition, *t*(57) = −2.39, *p* = 0.020, *d* = 0.3. There was no significant congruency effect for happy faces, *t*(57) = 0.01, *p* = 0.991, *d* < 0.01.

### Vertex Positive Potential

We performed the same analysis as for the N170. The 3 × 3 ANOVA showed no interaction, *F*(4,228) = 0.62, *p* = 0.651, ${\text{η}}_{\text{p}}^{2}$ = 0.01. The main effects did not reach significance, neither for odor, *F*(2,114) = 3.05, *p* = 0.051, ${\text{η}}_{\text{p}}^{2}$ = 0.05, nor expression, *F*(2,114) = 1.26, *p* = 0.288, ${\text{η}}_{\text{p}}^{2}$ = 0.02. The 2 × 2 ANOVA did not reach significance for interaction, *F*(1,57) = 1.28, *p* = 0.263, ${\text{η}}_{\text{p}}^{2}$ = 0.02, main effect of odor *F*(1,57) = 0.05, *p* = 0.826, ${\text{η}}_{\text{p}}^{2}$ < 0.01, or expression, *F*(1,57) = 0.11, *p* = 0.741, ${\text{η}}_{\text{p}}^{2}$ < 0.01.

### P2

Using the same design, we performed statistical tests on the occipital P2 ERP-component. The 3 × 3 ANOVA showed no interaction, *F*(4,228_GG_) = 0.18, *p* = 0.921, ${\text{η}}_{\text{p}}^{2}$ < 0.01. There was no statistically significant main effect of odor, *F*(2,114) = 0.98, *p* = 0.378, ${\text{η}}_{\text{p}}^{2}$ = 0.02, however, the main effect of expression was significant, *F*(2,114) = 3.78, *p* = 0.026, ${\text{η}}_{\text{p}}^{2}$ = 0.06. The 2 × 2 ANOVA showed no significant effects, interaction, *F*(1,57) = 0.10, *p* = 0.758, ${\text{η}}_{\text{p}}^{2}$ < 0.01, odor, *F*(1,57) = 0.29, *p* = 0.589, ${\text{η}}_{\text{p}}^{2}$ < 0.01, or expression, *F*(1,57) = 0.14, *p* = 0.712, ${\text{η}}_{\text{p}}^{2}$ < 0.01.

### Late Positive Potential

Lastly, we investigated whether evidence of odor-visual integration would also be present at later cortical processing stages; we conducted a similar analysis for the LPP component. The odor by expression. The 3 × 3 ANOVA, *F*(4,228) = 0.96, *p* = 0.432, ${\text{η}}_{\text{p}}^{2}$ = 0.02, for the interaction was not significant. There were no statistically significant main effects of odor, *F*(2,114) = 0.26, *p* = 0.770, ${\text{η}}_{\text{p}}^{2}$ < 0.01, or expression, *F*(2,114) = 1.96, *p* = 0.146, ${\text{η}}_{\text{p}}^{2}$ = 0.03. The 2 × 2 ANOVA on difference scores did not reach significance, for the odor by expression interaction, *F*(1,57) = 0.10, *p* = 0.753, ${\text{η}}_{\text{p}}^{2}$ < 0.01; main effect of odor, *F*(1,57) = 1.47, *p* = 0.231, ${\text{η}}_{\text{p}}^{2}$ = 0.03, or main effect of expression, *F*(1,57) = 0.56, *p* = 0.458, ${\text{η}}_{\text{p}}^{2}$ < 0.01. There was thus no evidence of emotional integration of odor-visual cues at this later stage.

## Discussion

Social processes are contingent on rapid, implicit integration of emotionally relevant information from different sensory channels, for example, understanding whether the facial emotional expression of another person is affected by the immediate environmental context. Few previous studies have investigated how arousal and valence affect the perception of facial expressions of emotion, and to our knowledge, a few have investigated these subjective properties in relation to cortical responses by such odor-visual integration. We used odors as contextual cues to examine their effects on behavioral and cortical responses to congruent or incongruent facial expressions.

Perhaps unsurprisingly, facial expressions were rated as more negatively valenced in an unpleasant odor context, and more positively valenced in a pleasant odor context, partially replicating the findings in Cook et al. (2017). Faces were also rated as more arousing overall when contextual odors were present. A novel finding was that odors affected emotional arousal ratings in an interactive way; specifically, the rated arousal of happy faces was lower in the unpleasant odor condition compared to the pleasant odor condition, perhaps suggesting an olfactory inhibition of incongruent emotional signals from the face stimuli. Such effects have been found for aversive odors in the Stroop task (Finkelmeyer et al., 2010). Our work partly replicates previous research findings for arousal ratings, such as Cook et al. (2017), however, this effect was not significant for disgusted expressions.

When it comes to the EEG results, the N170 was sensitive to the congruency of facial emotions and contextual odors. However, in contrast to the behavioral results, for the N170 this effect was for disgusted faces, in trials where an unpleasant odor provided the context, the N170 was weaker than in trials with a pleasant odor context. These results are congruent with the notion that for disgusted faces, a congruent odor may facilitate the processing of the faces while incongruent odors need more attentional resources. Previous research has shown that the N170 and its’ inverse VPP are generated by the same dipoles (Joyce and Rossion, 2005), in the ‘fusiform face area,’ and recent findings suggest that this area is also highly sensitive to cross-modal integration (Park et al., 2010; Gerdes et al., 2014). Interestingly, hypoactivation was observed for a congruent odor-visual stimulation of unpleasant odor and disgusted faces (Seubert et al., 2010a). This result indicates that, at least for disgusted expressions, congruent odor exposure may facilitate processing of the facial expression (Seubert et al., 2010a). These results might also be explained by differing habituation rates (i.e., disgusted faces paired with an incongruent pleasant odor habituate slower across multiple trials). Previously it has been shown that cortical activity in the N170 time range for aversively odor conditioned faces resisted habituation in contrast to the CS- (Steinberg et al., 2012). We were unable to replicate the effects of facial emotion observed in the VPP component by Leleu et al. (2015b). This could perhaps be explained by differences between studies in stimulus materials or of the chosen reference electrodes. It should be noted that our interaction effects on N170 amplitudes and perceived arousal were driven by different specific odor-visual combinations. Thus, more research is needed to firmly establish whether N170 amplitude modulations indeed underlie perceived arousal in this task, although we interpret our results as pointing in that direction.

We observed no odor effects in the earliest P1 stage, similar to most previous work (Rubin et al., 2012; Leleu et al., 2015b). In the present study, there were no odor effects in the mid-stage (P2) interval. Previously, one study found that an unpleasant odor, relative to neutral odors, increased the amplitude of the P2 to disgusted facial expressions (Leleu et al., 2015b). However, Rubin et al. (2012) could not show this effect in a study where human sweat was used as odor stimuli. We could not observe any odor-visual effects in the late-stage LPP component for either emotional faces or in combination with odors. Previous work interpreted the LPP as reflecting sustained attention to emotional (survival-relevant) information, as is demonstrated for emotional pictures (Olofsson et al., 2008). However, previous research has shown that emotional faces may also modulate the LPP, although, this effect appears to be much weaker than the effect of emotional scenes (Thom et al., 2014). In odor-visual interactions, previous studies show inconsistent results. In one study (Adolph et al., 2013), a decrease in LPP amplitude, while another found an increase in LPP amplitude (Rubin et al., 2012) in the context of sweat odor. Ours is the second study that could not find effects of emotional odors on LPP (Leleu et al., 2015b). The literature thus suggests no overall effect of odors at long-latency intervals, but points instead toward early stage integration around the N170 processing stage. As results are mixed, we suggest studies with higher power might be needed to detect subtle effects and resolve the present inconsistencies. As our study had a comparably large effective sample size (*n* = 58), our effects may provide a better estimation of true effects than comparable studies with less power.

The present study has several merits compared to previous research, such as a high-powered sample to detect effects that the previous literature indicated are small, and robust methods to ensure that olfactory intensity is balanced across odors and participants. There are also limitations. The ERP method requires multiple presentations of the stimuli to provide for a good signal-to-noise ratio. Because of limitations in the number of suitable images, each face was presented repeatedly in the EEG part of each block, and thus we cannot account for possible learning effects for example. However, the effect sizes for ratings in the present study are in line with previous research.

Olfactory influences on face perception may be highly adaptive. Prior research indicates that odors might reduce reaction times and enhance accuracy in emotion recognition (Seubert et al., 2010a,b). Besides these main effects, some studies showed that facial emotions are recognized both faster and more accurately in a congruent odor context (e.g., unpleasant odor paired with a disgusted facial expression (Leppanen and Hietanen, 2003; Leleu et al., 2015a). It was argued that odors reduce the amount of emotional information needed to recognize a congruent facial expression (Leleu et al., 2015a). These two types of effects may be explained by (1) affective odors increasing emotional arousal, which affects overall performance (Bensafi, 2002), (2) that there is cross-modal facilitation of processing for emotionally congruent information (Seubert et al., 2010a), or (3) differing rates of habituation (Steinberg et al., 2012). Overall, our ERP results fit better within the cross-modal framework, but our behavioral ratings indicate that both arousal-based and congruency-based effects may be present at different aspects of the emotional evaluation process. Further research is needed to clarify the specific experimental circumstances most likely to produce main effects vs. emotional interaction effects. As the odor effects observed in the present study and prior work are generally small, future work needs to have adequate statistical power to detect these subtle effects. More research is also needed to investigate the mechanisms behind odor effects. Several avenues are promising. For example, odors might guide attention to areas in faces that contain emotional information, or odors might delay adaptation or habituation processes for some facial expressions. In conclusion, our results suggest valenced odors may generate subtle emotional congruency effects when perceiving emotional facial expressions, as shown in the N170 ERP component.

## Author Contributions

ES, SW, HF, and JO contributed to the design, analysis, and drafting and interpretation of the present study. ES, MZ, and AW collected the data. All authors critically evaluated and revised the final manuscript.

## Conflict of Interest Statement

The authors declare that the research was conducted in the absence of any commercial or financial relationships that could be construed as a potential conflict of interest.

## Footnotes

1 www.epn.se/stockholm
