# Supplementary material for: Background Odors Modulate N170 ERP Component and Perception of Emotional Facial Stimuli
Source: Front Psychol. 2018 Jun 26;9:1000. doi: 10.3389/fpsyg.2018.01000 (PMC6029154; doi:10.3389/fpsyg.2018.01000)
Supplement: Supplementary file 1 [file Data_Sheet_1.DOCX]

Figures with congruent and incongruent odor/face conditions

As the present study contain 9 different conditions we have created figures that display the results for the conditions that were of main interest (congruent/incongruent odor/expression pairs). Here we display topographies, ERP waves and difference scores for the congruent and incongruent odor/faces conditions. Below, we add similar figures that display all conditions. Across all plots, 95% CIs were corrected as suggested by Morey (2008) to account for within subject designs.


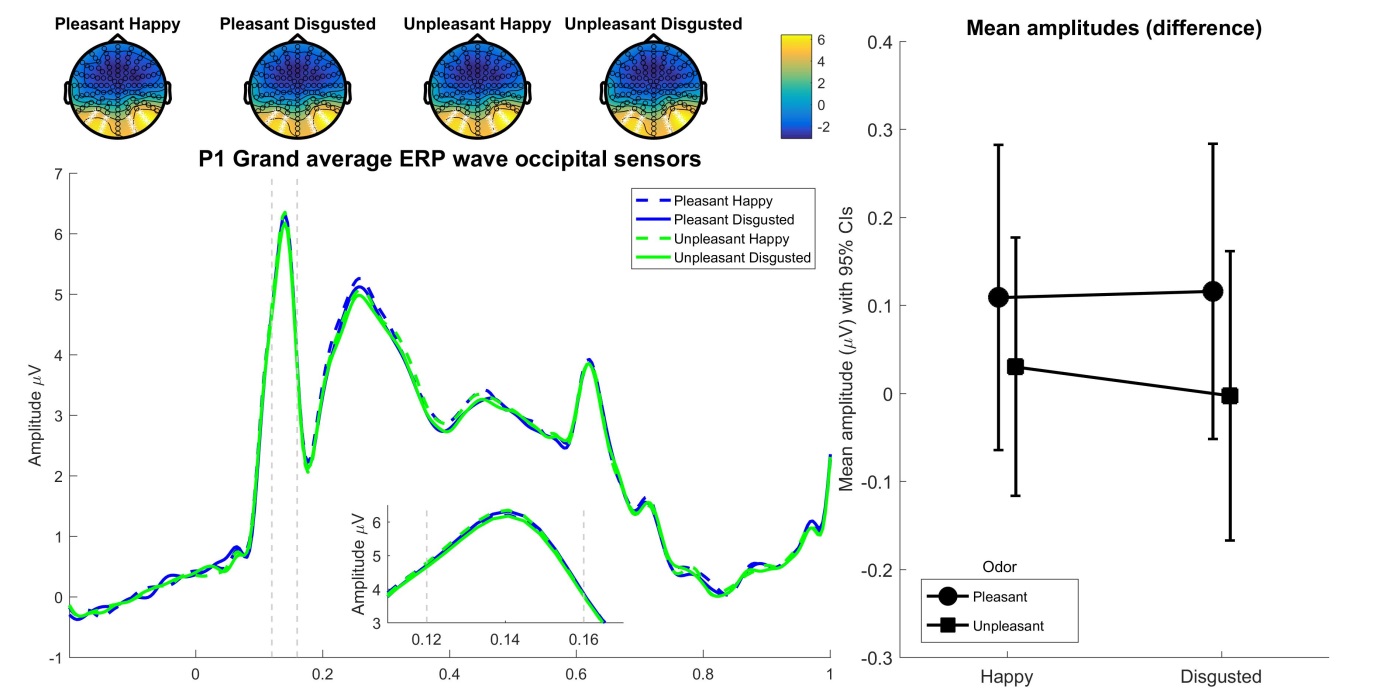
The top row depicts the raw topography of grand average (120-160 ms from stimulus onset) P1 for each odor-face condition. The left panel shows grand average ERP waves (of occipital sensors, highlighted in the top row) in each condition with an inset that zooms the peak of the P1 component. The right panel shows mean amplitudes and 95% confidence intervals around the mean amplitude difference between emotional and neutral conditions..

**
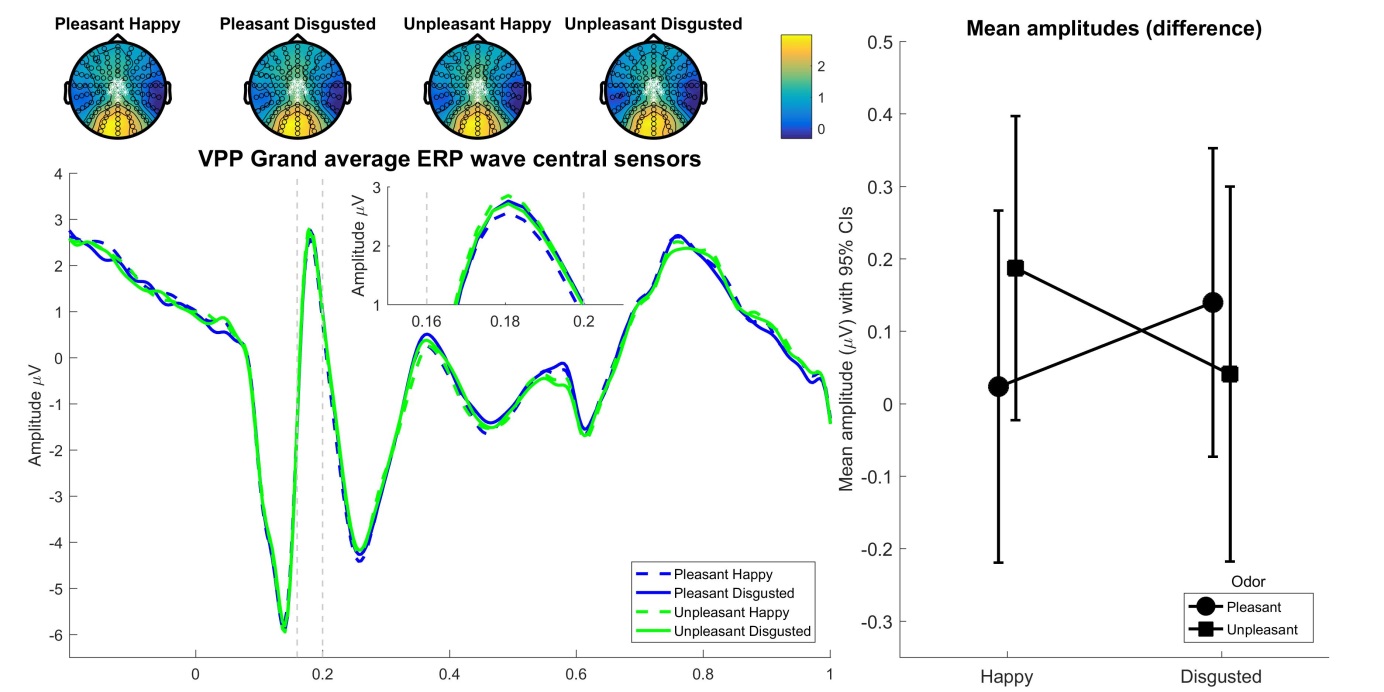
**

The top row shows the raw topography of grand average (160-200 ms from stimulus onset) VPP for each odor-face condition. The left panel shows grand average ERP waves (of central sensors, highlighted in the top row topographies) in each condition with an insert zooming in at the VPP peak. The right panel shows mean amplitudes and 95% confidence intervals around the mean amplitude difference between emotional and neutral conditions.


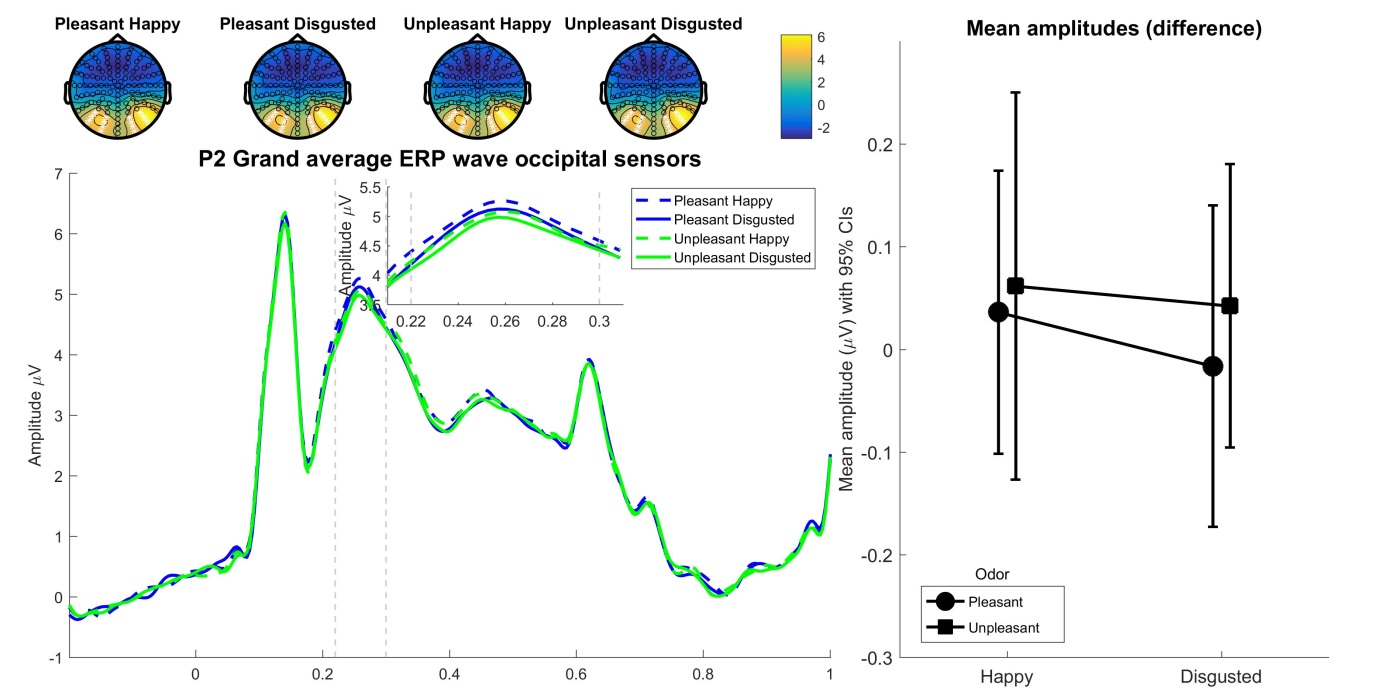


The top row shows the raw topography of grand average (220-300 ms from stimulus onset) P2 for each odor-face condition. The left panel shows grand average ERP waves (of occipital sensors, highlighted in the top row topographies) in each condition with an insert zooming in at the P2 peak. The right panel shows mean amplitudes and 95% confidence intervals around the mean amplitude difference between emotional and neutral conditions.


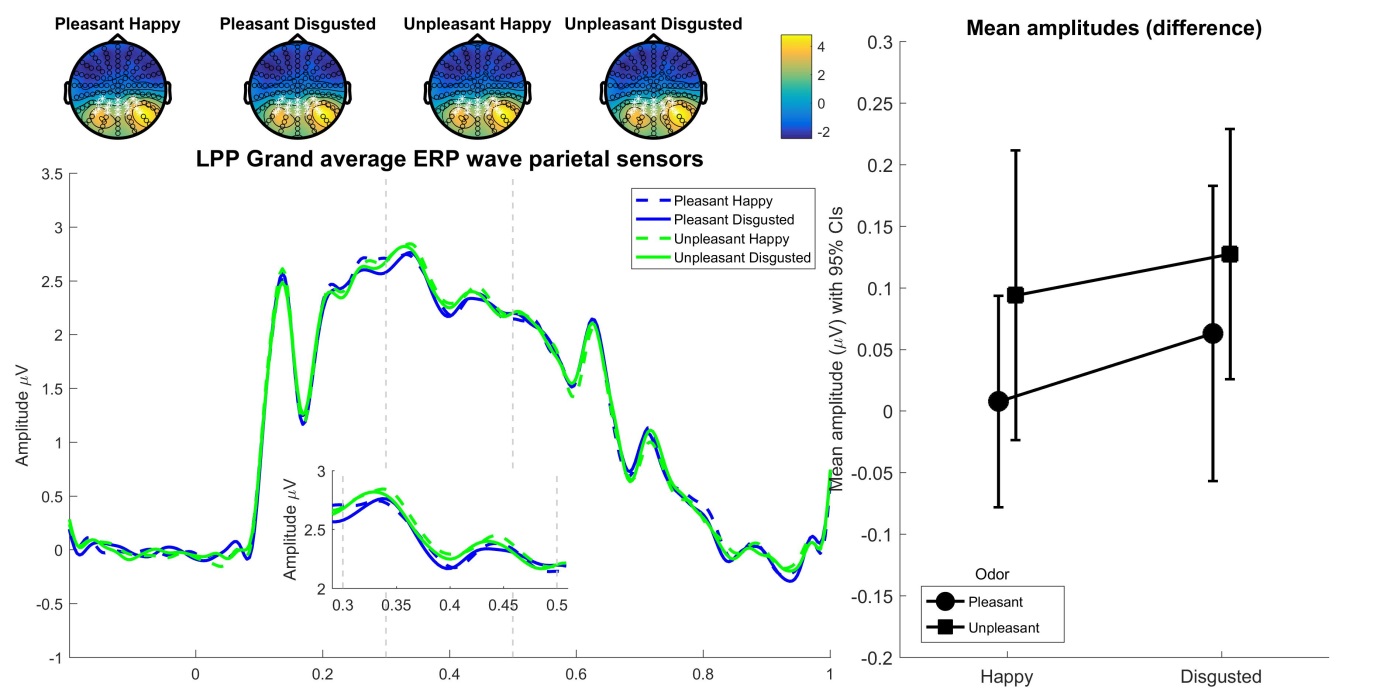


The top row shows the raw topography of grand average (300-500 ms from stimulus onset) LPP for each odor-face condition. The left panel shows grand average ERP waves (of parietal sensors, highlighted in the top row topographies) in each condition with an insert zooming in at the LPP time course. The right panel shows mean amplitudes and 95% confidence intervals around the mean amplitude difference between emotional and neutral conditions.

All conditions

Full experimental data can be found at following url (containing individual means for each participant and condition): https://figshare.com/s/252745f56a0f7b7115fc


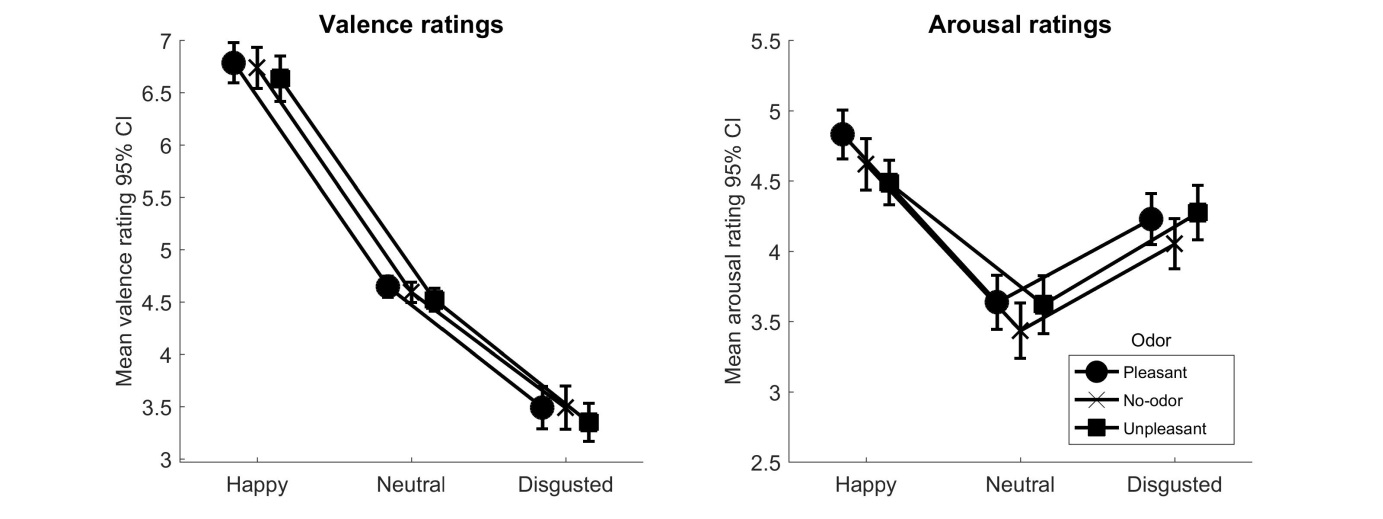


The panels show the rated valence (left) and arousal (right) for pleasant, no-odor and unpleasant odors, separately for happy, neutral, and disgusted facial expressions (means and 95% confidence intervals.


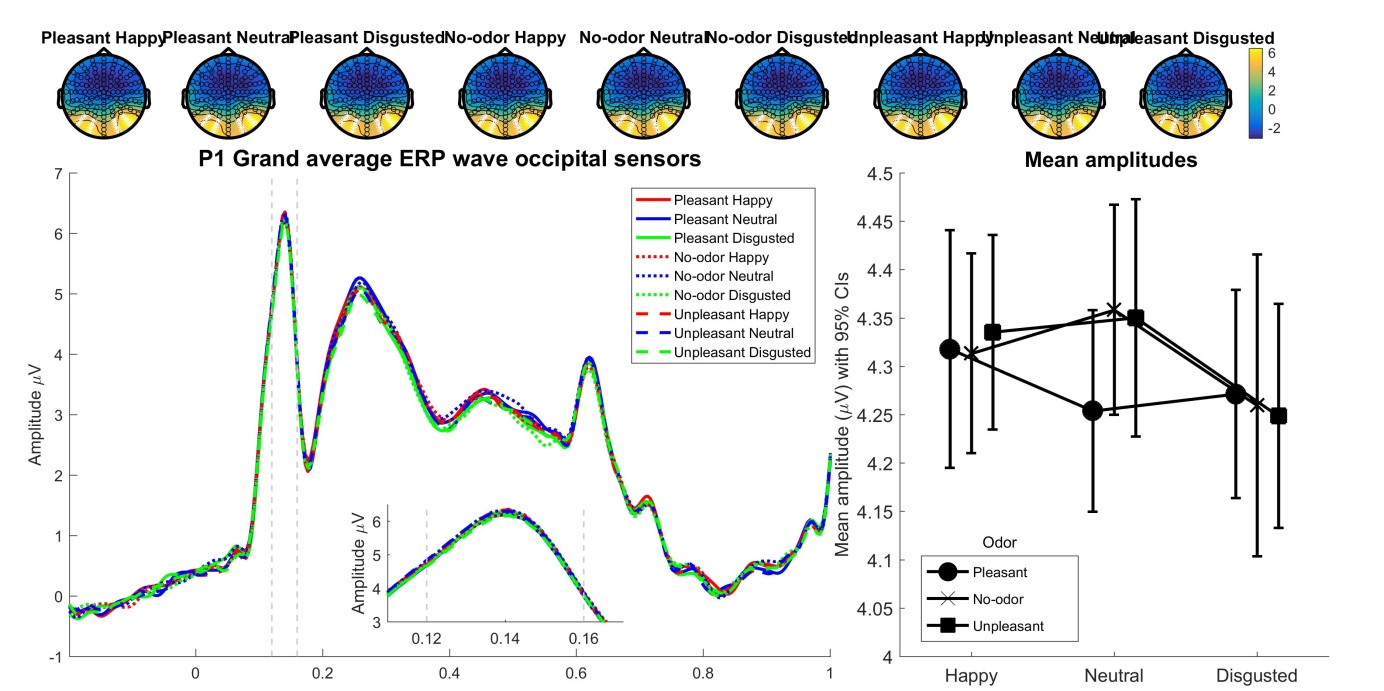


The top row depicts the raw topography of grand average (120-160 ms from stimulus onset) P1 for each odor-face condition. The left panel shows grand average ERP waves (of occipital sensors, highlighted in the top row) in each condition with an inset that zooms the peak of the P1 component. The right panel shows mean amplitudes and 95% confidence intervals around the mean amplitude.


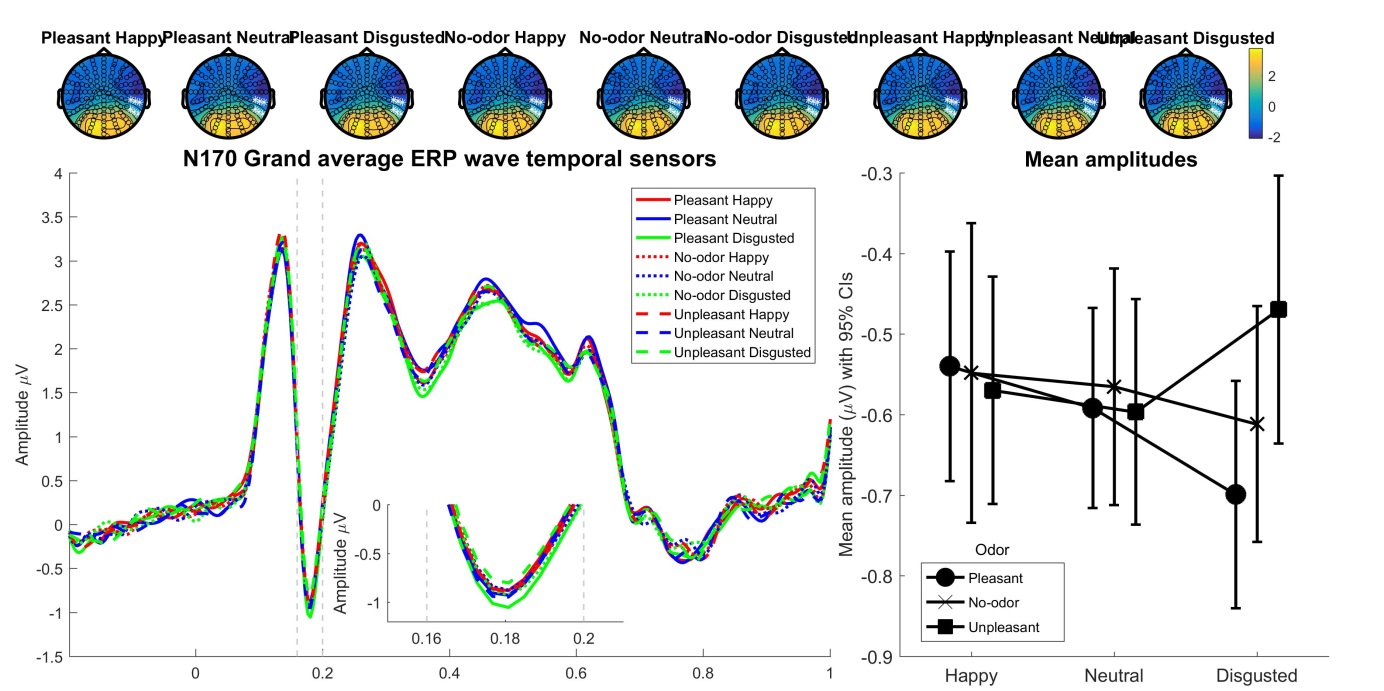


The top row shows the raw topography of grand average (160-200 ms from stimulus onset) N170 for each condition. The left panel shows grand average ERP waves (of right temporal sensors, highlighted in the top row topographies) in each condition with an insert zooming in at the N170 peak. The right panel shows mean amplitudes and 95% confidence intervals around the mean amplitude.


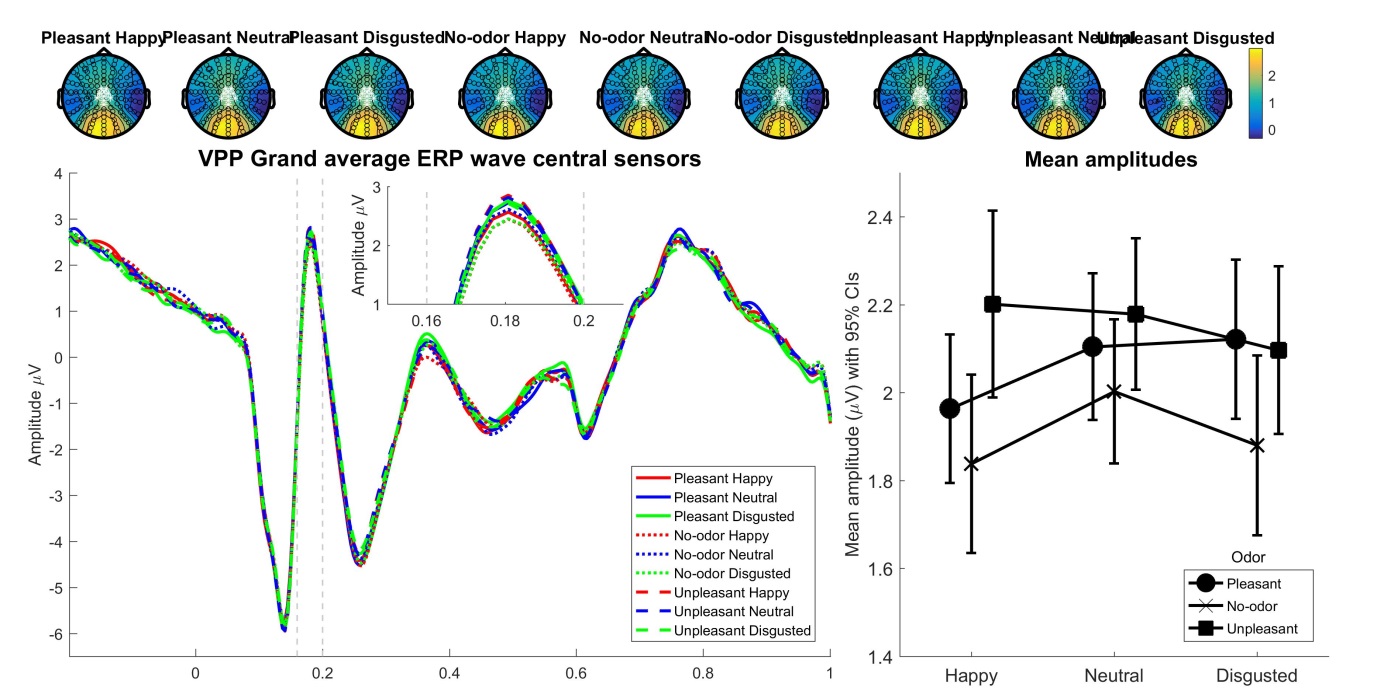


The top row shows the topography of grand average (160-200 ms from stimulus onset) VPP for each odor-face condition. The left panel shows grand average ERP waves (of central sensors, highlighted in the top row topographies) in each condition with an insert zooming in at the VPP peak. The right panel shows mean amplitudes and 95% confidence intervals around the mean amplitude for each condition.


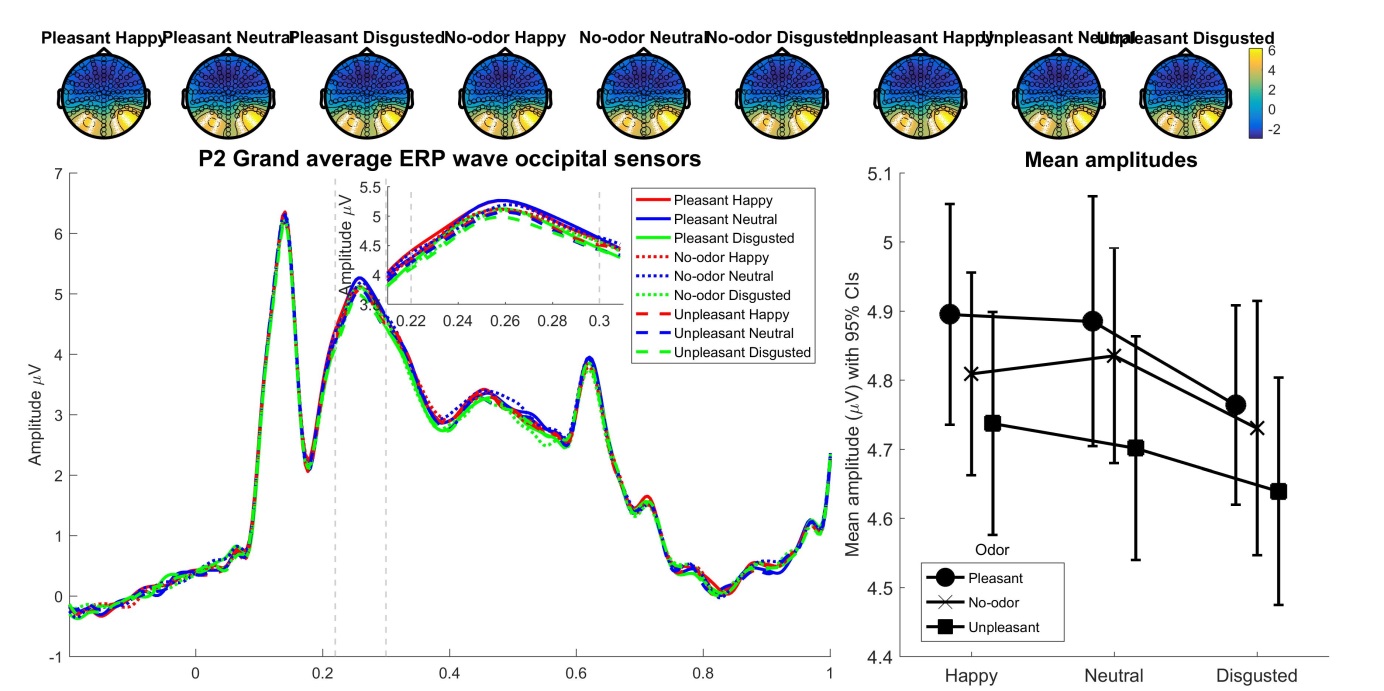


The top row shows the topography of grand average (220-300 ms from stimulus onset) P2 for each condition. The left panel shows grand average ERP waves (of occipital sensors, highlighted in the top row topographies) in each condition with an insert zooming in at the P2 peak. The right panel shows mean amplitudes and 95% confidence intervals around the mean amplitude.


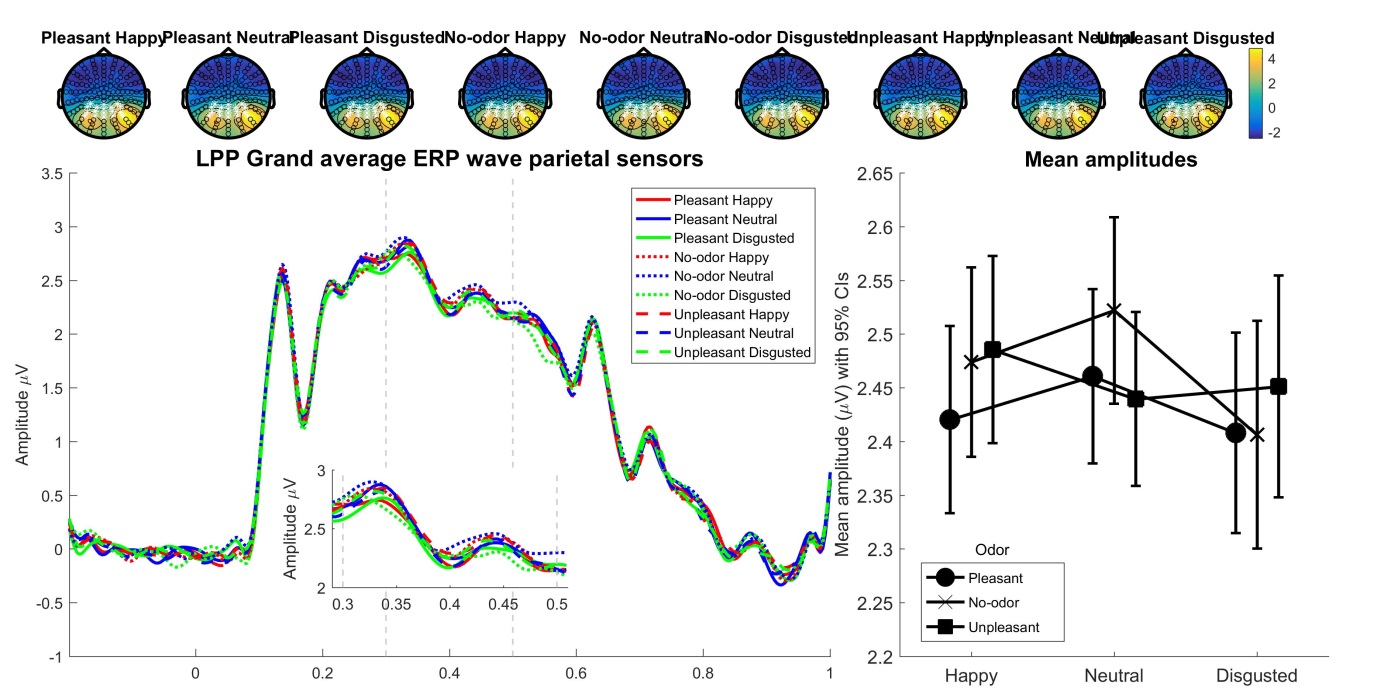


The top row shows the topography of grand average (300-500 ms from stimulus onset) LPP for each odor-face condition. The left panel shows grand average ERP waves (of parietal sensors, highlighted in the top row topographies) in each condition with an insert zooming in at the LPP time course. The right panel shows mean amplitudes and 95% confidence intervals around the mean amplitude.
